# Association between widespread pain and associated symptoms in patients with cirrhosis

**DOI:** 10.1097/HC9.0000000000000120

**Published:** 2023-04-14

**Authors:** Alexis Holman, Neehar D. Parikh, Zhe Zhao, Samantha Nikirk, Daniel J. Clauw, David A. Williams, Elliot B. Tapper

**Affiliations:** 1 Division of Rheumatology, University of Michigan, Ann Arbor, Michigan, USA; 2 Division of Gastroenterology and Hepatology, University of Michigan, Ann Arbor, Michigan, USA; 3 Anesthesiology Department, Chronic Pain and Fatigue Research Center, University of Michigan, Ann Arbor, Michigan, USA

## Abstract

Chronic pain is common in cirrhosis; however, the mechanistic classifications are not well characterized. Nociplastic pain, for instance, is associated with comorbid nonpain symptoms and responds to different treatments than nociceptive pain.

**Methods:**

We conducted a survey study in February 2022 of adult patients with cirrhosis at the University of Michigan (*N* = 238) to evaluate pain widespreadedness, associated nociplastic symptoms, and pain intensity.

**Findings:**

Pain and pain widespreadedness were associated with mood and cognitive disturbance, fatigue, sleep difficulty, and physical and social functioning. Patient-reported Outcomes, such as body maps, can be useful to phenotype patients for pain mechanisms.

## Graphical abstract

**Figure:**
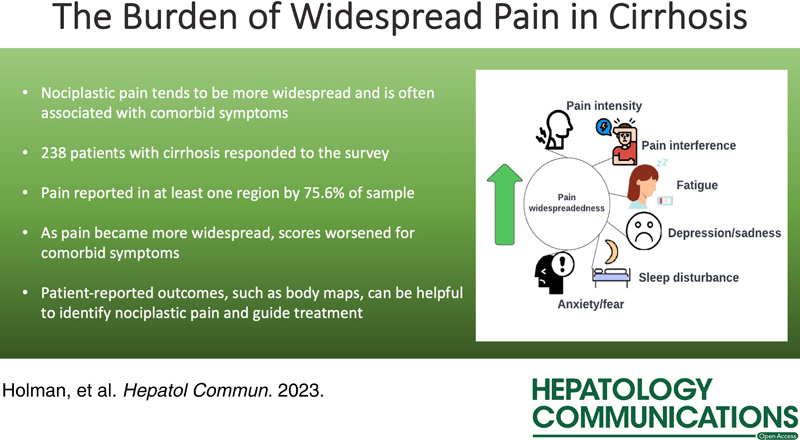

## INTRODUCTION

Chronic pain is common in cirrhosis, reported by up to 79% of patients.^1^ Although not well characterized in cirrhosis, it is likely that multiple mechanistic classifications are represented: for example, nociceptive pain (pain from tissue damage), neuropathic pain (pain from nervous system damage), and nociplastic pain, which describes central augmentation of nociception which tends to be more widespread (eg, fibromyalgia and irritable bowel syndrome).^2^ In addition, nociplastic pain is often associated with comorbid nonpain symptoms, such as fatigue, memory or concentration problems, mood disturbances, and sleep difficulties, that can negatively impact patient well-being.^2^,^3^ The recognition of pain classifications can be important, given that responsivity to treatment differs between classifications. Nociceptive pain tends to respond to NSAIDs, acetaminophen, opioids, injections, and surgery, whereas nociplastic pain preferentially responds to centrally acting analgesics (eg, tricyclics, serotonin and norepinephrine reuptake inhibitors, and gabapentinoids) with aggressive use of nonpharmacologic therapies.^2^ Thus, our primary focus in this study was to characterize the impact of nociplastic pain in individuals with cirrhosis.

## METHODS

### Sample

Surveys were sent to 2000 adult (aged 18 y or above) patients through email in February 2022. Participants had an established diagnosis of cirrhosis and were followed by a hepatologist at the University of Michigan. We excluded patients with liver transplants. Participants were paid 10 dollars for completing the survey. The study was approved by the University of Michigan Institutional Review Board, and all respondents provided informed consent.

### Measures

The widespreadedness of pain (a key feature of nociplastic pain), as well as associated nociplastic symptoms, was assessed using the American College of Rheumatology 2011 Fibromyalgia Survey Criteria.^4^ This survey includes the number of painful body regions (0–19; the Widespread Pain Index) and related symptoms such as problems with thinking, fatigue, and sleep difficulties (0–12; the Symptom Severity Index).^4^ This survey has been used to quantify centralized pain in other clinical populations,^5^ relates strongly to functional neuroimaging findings in nociplastic pain,^6^ and predicts pain and disability.^7^,^8^ Nonpain symptoms were assessed using the Patient-reported Outcome Measurement Information System 29-Item Profile, version 2 (PROMIS-29+2), which evaluates the following domains: Pain Interference, Physical Function, Fatigue, Anxiety, Depression, Sleep Disturbance, and Satisfaction with Participation in Social Roles, as well as cognitive function,^9^,^10^ and are scored using a T-score, where a score of 50 approximates the general population means with an SD of 10 points. Pain intensity was assessed with the PROMIS Pain Intensity 3a, a 3-item measure that assesses the worst and average pain in the past 7 days and the current pain.^11^ ANOVA was used to test the significance of the differences in each domain.

## RESULTS

### Sample characteristics

Overall, 238 patients responded to the survey (response rate 11.9%). Our study sample (*N*=238) was comprised of 40.8% of men with an average age of 58.8 years (Supplemental Table 1, http://links.lww.com/HC9/A245). Most (60%) had a college degree or more and were primarily White (89.5%). The commonest etiology of cirrhosis was NAFLD. Most were compensated, with 12.6% and 16% reporting ascites and HE, respectively. Overall, 21% were taking opioids. The most common pain region reported was the axial region (65.5%).

### Associated symptoms

As the number of positive pain regions increased from 0 to 4 or more, PROMIS29+2 scores increased for pain interference, depression/sadness, anxiety/fear, fatigue, and sleep disturbance, where higher scores reflect worse symptoms (Table 1). Likewise, as the number of pain regions increased from 0 to 4 or more, PROMIS29+2 scores decreased for cognitive function, physical function, and ability to participate in social roles/activities, where lower scores reflect worse functioning.

**TABLE 1:** Relationship between widespread pain index and associated symptom scores

		No. pain regions present				
	PROMIS 29+2 category	0 regions (24.3%, n=58)	1 region (16.3%, n=39)	2–3 regions (33.2%, n=79)	4 or more regions (26.0%, n=62)	*p*
Higher scores are worse	Symptoms	Mean + SD				
	Pain Interference	46.15±5.40	52.56±3.84	56.79±3.07	60.99±2.78	<0.001
	Depression/Sadness	47.17±4.94	50.50±4.34	54.09±3.69	56.46±3.49	<0.001
	Anxiety/Fear	49.08±4.74	52.25±4.15	54.86±3.79	58.23±3.37	<0.001
	Fatigue	49.74±3.02	54.27±2.67	59.40±2.74	62.31±2.83	<0.001
	Sleep Disturbance	49.31±3.61	52.00±3.56	54.64±3.37	56.70±3.44	<0.001
Lower scores are worse	Functions	Mean + SD				
	Cognitive Function	54.49±6.32	51.69±5.85	50.09±5.76	47.58±5.82	<0.001
	Physical Function	50.74±5.46	45.44±3.86	41.55±3.31	38.49±3.15	<0.001
	Ability to Participate in Social Roles/Activities	55.86±4.01	51.12±2.98	46.32±2.78	44.98±2.80	<0.001
Higher worse	PAIN Intensity PROMIS3a	41.58±4.86	49.70±4.04	54.55±3.76	59.53±3.77	<0.001

Abbreviation: PROMIS, Patient-reported Outcome Measurement Information System.

### Pain intensity

As the number of pain regions present increased from 0 to 4 or more, Pain Intensity PROMIS3a scores increased, where higher scores reflect worse pain intensity (Table 1).

### Pain region

There was no consistent association between pain region and associated symptoms or pain intensity (Supplemental Table 2, http://links.lww.com/HC9/A246).

## DISCUSSION

Here, we find that pain and pain widespreadedness are associated with mood and cognitive disturbance, fatigue, sleep difficulty, and physical and social functioning. Pain in a specific region did not seem to affect any domain particularly. Overall, these results suggest that nociplastic pain may be a clinically significant contributor to the burden of chronic pain in patients with cirrhosis. Patient-reported Outcomes (PROs) like body maps can be useful to phenotype these patients for pain mechanism and help predict treatment responses.

Identifying patients with features of nociplastic pain is important because first-line treatment is nonpharmacologic, incorporating patient education, self-management strategies, lifestyle interventions (eg, exercise/diet optimization, healthy sleep habits, stress relief, and maintenance of occupational/social involvement), and psychological treatments (eg, cognitive behavioral therapy), which can be useful in patients with cirrhosis, for whom pharmacologic therapy is complex.^2^,^12^

In addition, PROs can identify patients with characteristics of nociplastic pain and help to predict the presence of nonpain symptoms that can guide therapy. For instance, in addition to nonpharmacologic therapy, low-dose tricyclic compounds can address multiple symptoms and are first-line for nociplastic pain.^3^ Serotonin-norepinephrine reuptake inhibitors can be used for accompanying depression or fatigue, and gabapentinoids can be used for anxiety or sleep disturbance.^3^

Similarly, PROs can predict treatment response. Conventional analgesics (eg, NSAIDs and acetaminophen) are not generally useful for nociplastic pain, and opioid use is strongly discouraged.^2^,^3^ In fact, higher fibromyalgia survey scores have been correlated with increased postoperative opioid consumption, suggesting decreased responsiveness to these medications.^13^ There is evidence that this type of pain might be worsened by opioids through opioid-induced hyperalgesia.^14^

Our study has various limitations, significantly the small sample size and primarily White, educated sample, which restricts the generalizability of our results.

Overall, nociplastic pain seems to be an important pain phenotype in patients with cirrhosis, for which treatment centers on nonpharmacologic therapies, as well as the improvement of nonpain symptoms and functioning.^2^,^3^ PROs, such as Fibromyalgia surveys and body maps, can be essential for identifying nociplastic pain and guiding treatment. One promising area of future research is to develop additional validated surveys tailored for use in patients with cirrhosis.

## Supplementary Material

SUPPLEMENTARY MATERIAL
