# Supplementary material for: Association between widespread pain and associated symptoms in patients with cirrhosis
Source: Hepatol Commun. 2023 Apr 14;7(5):e0120. doi: 10.1097/HC9.0000000000000120 (PMC10109455; doi:10.1097/HC9.0000000000000120)
Supplement: SUPPLEMENTARY MATERIAL [file hc9-7-e0120-s001.docx]

**Supplementary Table 1. Demographics of survey respondents (*N* = 238)**

| **Descriptive** | Value |
| --- | --- |
| **Gender** |  |
| *Men* | 40.8% (n = 97) |
| *Women* | 58.8% (n = 140) |
| *Other* | 0.42% (n = 1) |
| **Age in years (M, SD, range)** | 58.8, 12.4, 52-67.8 |
| *Education* |  |
| *Less than college* | 13.8% (n = 33) |
| *Some college, no degree* | 25.6% (n = 61) |
| *College degree (Associate, Bachelor’s)* | 36.5% (n = 104) |
| *Greater than college degree (Master’s, Professional, Doctoral)* | 23.5% (n = 56) |
| *Unknown* | 0.4% (n = 1) |
| **Race** |  |
| *White* | 89.5% (n = 213) |
| *African American* | 4.6% (n = 11) |
| *Other (Asian, American Indian, Native Hawaiian or Pacific Islander)* | 4.2% (n = 9) |
| *Unknown or not reported* | 4.2% (n = 10) |
| **Etiology of cirrhosis** |  |
| *Non-alcoholic fatty liver disease* | 47.5% (n = 113) |
| *Alcohol* | 17.2% (n = 41) |
| *Hepatitis C* | 16.0% (n = 38) |
| *Other* | 30.2% (n = 72) |
| **Ascites** | 12.6% (n = 30) |
| **Hepatic encephalopathy** | 16.0% (n = 38) |
| **Opioid use (% on opioids)** | 21.0% (n = 50) |
| **Pain region reported** |  |
| *Left upper region* | 35.7% (n = 85) |
| *Right upper region* | 31.5% (n = 75) |
| *Left lower region* | 40.3% (n = 96) |
| *Right lower region* | 51.3% (n = 122) |
| *Axial region* | 65.5% (n = 156) |
