# Supplementary material for: Association between widespread pain and associated symptoms in patients with cirrhosis
Source: Hepatol Commun. 2023 Apr 14;7(5):e0120. doi: 10.1097/HC9.0000000000000120 (PMC10109455; doi:10.1097/HC9.0000000000000120)
Supplement: SUPPLEMENTARY MATERIAL [file hc9-7-e0120-s002.docx]

| **Outcome Measure** | **WPI Region** | **Beta Estimate** | **P-Value** |
| --- | --- | --- | --- |
| PROMIS29+2 v2.1 - Pain Interference | Left upper | 1.3 | 0.4 |
|  | Right upper | 4.09 | 0.008 |
|  | Left lower | 4.78 | 0.002 |
|  | Right lower | -0.45 | 0.8 |
|  | Axial | 6.55 | 0.000006 |
| PROMIS29+2 v2.1 - Cognitive Function | Left upper | -1.38 | 0.3 |
|  | Right upper | -0.15 | 0.9 |
|  | Left lower | -1.98 | 0.1 |
|  | Right lower | -0.83 | 0.5 |
|  | Axial | -3.1 | 0.01 |
| PROMIS29+2 v2.1 - Depression/Sadness | Left upper | 0.31 | 0.9 |
|  | Right upper | 2.64 | 0.1 |
|  | Left lower | 2.47 | 0.1 |
|  | Right lower | 0.86 | 0.6 |
|  | Axial | 3.66 | 0.02 |
| PROMIS29+2 v2.1 - Ability to Participate in Social Roles/Activities | Left upper | 0.77 | 0.6 |
|  | Right upper | -3.07 | 0.06 |
|  | Left lower | -3.24 | 0.05 |
|  | Right lower | 0.93 | 0.6 |
|  | Axial | -7.59 | 0.000001 |
| PROMIS29+2 v2.1 - Anxiety/Fear | Left upper | 0.3 | 0.9 |
|  | Right upper | 1.44 | 0.4 |
|  | Left lower | 2.72 | 0.1 |
|  | Right lower | 0.75 | 0.7 |
|  | Axial | 4.42 | 0.009 |
| PROMIS29+2 v2.1 - Fatigue | Left upper | -0.02 | 1 |
|  | Right upper | 2.09 | 0.2 |
|  | Left lower | 4.74 | 0.006 |
|  | Right lower | -0.48 | 0.8 |
|  | Axial | 7.45 | 0.000007 |
| PROMIS29+2 v2.1 - Physical Function | Left upper | -0.17 | 0.9 |
|  | Right upper | -3.58 | 0.01 |
|  | Left lower | -4.19 | 0.004 |
|  | Right lower | 0.3 | 0.8 |
|  | Axial | -5.67 | 0.00005 |
| PROMIS29+2 v2.1 - Sleep Disturbance | Left upper | 1.06 | 0.5 |
|  | Right upper | 1.86 | 0.2 |
|  | Left lower | 3.79 | 0.02 |
|  | Right lower | -3.29 | 0.03 |
|  | Axial | 5.17 | 0.0007 |
| PAIN PROMIS3a v2.0 - Pain | Left upper | 1.68 | 0.3 |
|  | Right upper | 4.72 | 0.006 |
|  | Left lower | 2.62 | 0.1 |
|  | Right lower | 1.39 | 0.4 |
|  | Axial | 8.78 | 0.0000001 |

**Supplementary Table 2. Association between pain region and outcome measures**

WPI = Widespread Pain Index
